# A comparison of Glucagon-like peptide-1 receptor agonists on weight change, side effects, and quality of life in Kuwait

**DOI:** 10.3389/fnut.2025.1590379

**Published:** 2025-05-19

**Authors:** Saleh Alali, Wed Al-Otaibi, Karen Ashodian, Nour Achour, Aishah Almulla, Hessa Mutlaq

**Affiliations:** Nutrition and Food Science Department, Kuwait University, Kuwait City, Kuwait

**Keywords:** Glucagon-like peptide-1 receptor agonists, Semaglutide, Liraglutide, Tirzepatide, weight-loss, Kuwait

## Abstract

**Introduction:**

An estimated 74.6% of Kuwaiti adults are classified as overweight or obese. As a result, Glucagon-like peptide 1 receptor agonists (GLP-1 RA) injections such as, Semaglutide, Liraglutide, and Tirzepatide have become an increasingly popular intervention for weight loss. However, no research has been published on the use of GLP-1 RA injections in Kuwait.

**Methods:**

A cross-sectional study using an online survey was conducted between February and May 2024. Adults in Kuwait who have taken or are currently on GLP-1 RA injections were recruited. The questionnaire collected demographic and clinical data, including weight-loss, side-effects, and quality of life.

**Results:**

In this sample of *N* = 486, Tirzepatide users (*N* = 132; 27.8%) reported significantly higher monthly and annual weight-loss (4.76 ± 2.82 kg/month; 8.48 ± 4.04 kg/year), and greater satisfaction (88%) and improved quality of life (60%) compared to both Semaglutide (*N* = 181; 37.2%) and Liraglutide (*N* = 152; 31.3%), respectively. Side-effects varied: Tirzepatide users experienced more belching while Liraglutide reported higher anxiety levels and switching GLP-1 RA medication. No significant differences were found in BMI, following a diet, or compliance to treatment across the different GLP-1 RA groups.

**Discussion:**

This study highlights the varying effectiveness and tolerability of GLP-1 RAs for weight loss in Kuwait, with Tirzepatide showing the highest satisfaction and weight loss. Liraglutide users experienced more anxiety and medication switches, while Semaglutide users had moderate outcomes. Despite their benefits, GLP-1 RAs face challenges in long-term adherence due to side effects and cost.

## 1 Introduction

Non-communicable diseases (NCD) are the leading cause of mortality globally and are responsible for ~41 million deaths annually (1). Obesity is a global public health concern and is linked to an increased risk of developing NCDs such as type 2 diabetes, cardiovascular disease, and certain types of cancer. The public health situation in Kuwait, a Gulf Cooperation Council (GCC) member, is particularly alarming where an estimated 74.6% of adults are obese or overweight (2). The high incidence of obesity places a significant burden on the Kuwait healthcare system and stresses the need for effective healthcare interventions to address this epidemic. Sleeve gastrectomies, Roux-en-Y gastric bypasses and other surgical interventions are common weight-loss strategies used in Kuwait (3). However, recently, the use of Glucagon-Like Peptide-1 receptor agonists (GLP-1RAs) has gained significant attention for their effectiveness in weight-loss and diabetes management and are increasingly being used as an alternative to invasive surgeries (4).

The use of GLP- 1 RAs in injectable and oral formulations is suspected to increase as research continues to demonstrate its promising therapeutic potential beyond diabetes. Findings from clinical studies confirm the efficacy of GLP-1 RAs in weight management, platelet aggregation reduction, and neuroprotection (5–8). GLP-1 RAs were developed as an alternative to insulin for diabetes treatment. However, the substantial weight loss observed with GLP-1 RAs surged its off-label prescription for obesity and weight management, leading to price increases, global shortages, and creation of black markets to supply demand (9, 10).

Currently, GLP-1 RAs are available with a prescription in Kuwait. However, no studies have been published on their use and availability. Therefore, clinical eligibility requirements for prescription such as, a minimum BMI or threshold hemoglobin A1C is not clear. The lack of standardized prescription guidelines and inconsistent availability impacts patient access and treatment outcomes. One pre-filled injection of Ozempic is currently priced at 35 KWD (113 USD) which is nearly 10-fold less than its out-of-pocket price in the U.S (965 USD). GLP-1 RAs are also available in government healthcare facilities for free, but medication shortages and long wait times remain issues that drive patients to visit private clinics instead.

Ozempic^®^ and Wegovy^®^ (Semaglutide) and Saxenda^®^ (Liraglutide) are GLP-1 RAs, while Mounjaro^®^ (Tirzepatide) is a dual GLP-1 RA and gastric inhibitory peptide (GIP) receptor agonist. GLP-1 receptor agonist medications function similarly by reducing blood glucose levels, increasing satiety, and delaying gastric emptying (11, 12). Specifically, in response to carbohydrate intake, GLP-1 hormone is released from enteroendocrine cells and initiates a cascade of reactions including, insulin secretion, slowing gastric emptying, and regulating appetite. GLP-1 receptor agonists mimic GLP-1 by binding to the same active sites on GLP-1 receptors prolonging the activation of the same pathways without energy intake (13). Current clinical recommendations for Semaglutide involve a once-weekly injection of doses ranging from 0.25 mg to 2.4 mg (14). Liraglutides doses start at 0.60 mg and gradually increase to 3.0 mg daily injections (15). Finally, Tirzepatide doses start at 2.5 mg once-weekly injections and gradually increase to 15 mg (16).

Although the dosing regimens are well established, side-effects and impacts on quality of life across different GLP-1 RA's have yet to be fully elucidated. Gastrointestinal discomfort like, nausea and constipation are the most common adverse side-effects reported with injections (17). The severity of side-effects has been shown to be dose dependent impacting tolerability and adherence to treatment. Rare but concerning side-effects reported include, pancreatitis and bowel obstruction, and long-term safety remains unclear. Weight-loss is associated with significant improvements in both physical and mental quality of life metrics (18). However, the extent to which side-effects from GLP-1 RAs may cancel out quality of life improvements associated with weight loss is unclear.

Overall, differences in side-effects associated with GLP-1 RAs injections and potential impact on quality of life requires further study, especially in Kuwait. Clinical studies have established the efficacy of GLP-1 RAs in reducing weight and blood sugar levels. The transformative results seen with GLP-1 RAs have increased their use for obesity and offers a less invasive option compared to bariatric surgeries (19). However, most studies are comparing GLP-1 RAs to a placebo or insulin injections and rarely other GLP-1 RAs (20). Comparing GLP-1 RAs and evaluating their differences is needed to inform both healthcare professionals and patients to help them decide on the best treatment option for their needs. Currently no studies have been published on the use, side-effects, and efficacy of different GLP-1 RA injections in Kuwait. To address this gap in literature, this study aims to evaluate and compare the differences of Semaglutide, Liraglutide, and Tirzepatide on weight-loss, side-effects, and quality of life among patients in Kuwait, using a mixed-method approach. Additionally, it aims to inform our understanding of patient experiences and tolerability of these medications in Kuwait.

## 2 Materials and methods

To evaluate the differences in weight loss, side effects, and quality of life among users of three GLP-1 RA injection medications, Semaglutide, Liraglutide, and Tirzepatide, a cross-sectional study was designed using an online questionnaire. The 38-item questionnaire was administered through Microsoft Forms to individuals living in Kuwait who were prescribed GLP-1 medications for weight loss. Participants were recruited between Feb 2024 and May 2024, mainly in weight-loss clinics and through social media applications such as X (Twitter) and Instagram. The questionnaire was available in both English and Arabic to cater to a wider population. The inclusion criteria were adults in Kuwait prescribed GLP-1 RA injections for weight loss.

The questionnaire consisted of a combination of both quantitative and qualitative measures to provide a comprehensive analysis of participants' experiences with this relatively new weight-loss method. The survey included seven demographic items to collect background information, and five items were used to gather current clinical data such as height, weight, and current health status. Additionally, three items were used to understand the motivation and reasoning behind opting for GLP-1 medications as a weight-loss method. These questions listed common reasons such as “Health Problems” and “Nothing else worked,” while also allowing participants to provide written responses. Previous methods of weight loss and motivation to lose weight were also assessed to gain a comprehensive understanding of participants' reasoning for taking GLP-1 RA injections.

The survey collected information on the type, dose, and duration of use for different GLP-1 RA injection medications. Participants were then grouped based on the type of GLP-1 RA used.

Weight loss in a month and annually was measured as a categorical variable (1–5 kg, 5–10 kg, and 10+ kg). Subsequently, these categories were coded as continuous variables by assigning the midpoint value to each response (1–5 kg = 2.5 kg, 5–10 kg = 7.5 kg, and over 10 kg = 12.5 kg). Questions regarding adherence to a specific diet, compliance with the prescribed treatment schedule, and consulting a dietitian during treatment were measured as categorical variables to analyze whether these factors influenced treatment outcomes across GLP-1 RA medication groups.

Side effects, both during and after treatment, were evaluated using six items. In this section, participants were allowed to choose from a list of common side effects associated with GLP-1 injections or provide written responses. The questions in this section also addressed specifics, including the concentrations associated with side effects and whether side effects led participants to stop treatment or change the type of GLP-1 used.

Goal achievement, likelihood of recommending the medication, and effects on quality of life and psychological health were measured as categorical variables (Yes or No). Satisfaction levels were measured on a 1–3 Likert Scale, where a higher score was given to “Very Satisfied” and lower scores to “Very Dissatisfied.” Finally, two items were used to measure the number of participants who had stopped treatment along with their reasons for discontinuation. Common reasons such as “Cost” and “Side effects” were listed, as well as the option to provide a written response.

### 2.1 Statistical analysis

Statistical analysis was conducted using IBM SPSS Statistics version 29.0.0.0. One-way ANOVA was used to compare the mean differences in BMI, weight loss, and other measures across the three GLP-1 RA drug groups: Semaglutide, Liraglutide, and Tirzepatide. Chi-squared tests were used to compare differences in categorical variables across groups. Statistical significance was determined at *p-values* < 0.05.

## 3 Results

### 3.1 Demographic characteristics

The survey collected 545 responses in total. Table 1 presents demographic and clinical characteristics of all participants. Most participants were female 89% while only 11% were male. 81% identified as Kuwaiti nationals. 52.7% of participants were in the 25–40 age groups, followed by 30.6% that are over the age of 40, and lastly 16.7% were between the ages of 18–25 years old. 61.7% of participants were married and 71.7% have obtained at least a bachelor's degree. Lastly, 72.5% of participants were employed and a third (33.6%) reported a monthly income between 1,000–1,500 KWD.

**Table 1:** Demographic and clinical characteristics of patients on GLP-1 RA medications for weight loss in Kuwait (*n* = 545).

**Characteristic**	***N* (%)**
**Gender**	
Female	483 (89)
Male	61 (11)
Missing	1 (0.2)
**Age (years)**	
18–25	91 (16.7)
25–40	287 (52.7)
Over 40	167 (30.6)
**Nationality**	
Kuwaiti	441 (81)
Non-Kuwaiti	104 (19)
**Marital status**	
Married	336 (61.7)
Single	208 (38.2)
Missing	1 (0.2)
**Education**	
High school degree or less	65 (11.9)
Bachelor's degree	391 (71.7)
Higher education (Master or Doctorate degree)	88 (16.1)
Missing	1 (0.2)
**Employment**	
Working (paid employee)	395 (72.5)
Retired	78 (14.3)
Student	70 (12.8)
Missing	2 (0.4)
**Monthly income (KWD)**	
Lower than 500	68 (12.5)
500–1,000	132 (24.2)
1,000–1,500	183 (33.6)
Higher than 1,500	158 (29)
Missing	4 (0.7)
**Comorbidity**	
Hypertension	28
Type II diabetes mellitus	37
Chronic diseases	23
Cardiovascular disease	2
Other	81
**GLP-1 RA medication used (*****n*** = **486**^a^**)**	
Semaglutide (Ozempic or Wegovy)	181 (37.2)
Liraglutide (Saxenda or Victoza)	152 (31.3)
Tirzepatide (Mounjaro)	135 (27.8)
Two medications	10 (2)
All of the above	8 (1.6)
	**Mean** ±**SD**
Current BMI (kg/m2) (*N* = 477^b^)	29.29 ± 4.90

This summarizes demographic and clinical characteristics of patients that have taken or are currently taking GLP-1 RA medication for weight loss. Out of 545 participants, 89% were female while 11% were male. The largest age group of participants, 52.7%, were between 25–40 years old, while the least common, 16.7%, was 18–20 years old. 59 participants did not complete the survey and their answers were excluded from the analysis. Out of the 486 participants remaining 37.2% were on or have taken Semaglutide, 31.3 on Liraglutide, and lastly 27.8% on Tirzepatide. For the purpose of this study participants who have taken more than one medication or all types of GLP-1 RA were excluded from the study.

^a^Missing data for n = 59. ^b^Missing BMI for n = 9.

In this cohort, the most common comorbidity was Type II Diabetes (*n* =37) followed by hypertension (*n* = 28), and lastly chronic diseases (*n* = 23). Eighty one participants responded with other conditions, with the most common responses collected being, hypothyroidism, PCOS, and insulin resistance.

A total of 59 participants did not indicate which GLP-1 RA medication they used, leading to the exclusion of their responses from the analysis. Out of the 486 participants remaining, 37.2% were on or have taken Semaglutide, 31.3% on Liraglutide, and lastly 27.8% on Tirzepatide. Participants who had taken multiple GLP-1 RA medications were excluded from the study. The mean current BMI was 29.29 kg/m^2^ with a standard deviation of 4.90.

### 3.2 Weight and treatment outcomes

Table 2 includes clinical characteristics of the 459 participants across the three medication groups. Each group had *n* =3 missing entries for either weight or height and BMI data was missing. The mean BMI for the Tirzepatide group (29.76 ± 5.45 kg/m^2^) was slightly higher when compared to both groups, while the Semaglutide group had the lowest mean BMI at (28.91 ± 4.39 kg/m^2^). However, no significant differences were found in BMI across the three groups.

**Table 2:** Comparison of demographic and clinical characteristics between GLP-RA medication groups *n* = 459^a^.

**Clinical measures**	**Semaglutide (Ozempic or Wegovy) *n* = 178^b^**	**Liraglutide (Saxenda or Victoza) *n* = 149^c^**	**Tirzepatide (Mounjaro) *n* = 132^d^**	**Total *n* = 459**	***P*−*value*^e^**
**Mean** ±**SD**					
BMI	28.91 ± 4.39	29.15 ± 4.91	29.76 ± 5.45	29.22 ± 4.91	0.351^f^
Weight loss per month (kg) *n* = 413 Missing = 55	3.75 ± 2.21 (*n* =163)	4.00 ± 2.41 *N* = 123	4.76 ± 2.82 *N* = 127	4.14 ± 2.50 *N* = 413	0.002^f*^
Weight loss per year (kg)	7.14 ± 4.04 *N* = 170	6.18 ± 3.83 *N* = 147	8.48 ± 4.04 *N* = 131	7.21 ± 4.07 *N* = 448	< 0.001^f*^
Duration of use	*N* = 180	*N* = 152	*N* = 135	*N* = 467	
< 1 year *N* (%)	149 (82.8)	108 (71.1)	119 (88.1)	376 (80.5)	0.002^g*^
1–2 years *N* (%)	24 (13.3)	29 (19.1)	14 (10.4)	67 (14.3)	
> 2 years *N* (%)	7 (3.9)	15 (10.4)	2 (1.5)	24 (5.1)	
Dietary adherence	57 (31.5)	45 (29.6)	54 (40)	156 (33.3)	0.140^g^
Side effects	71 (39.2)	82 (53.9)	64 (47.4)	212	0.051^g^
Consulted with a dietitian	105 (58.7)	69 (46.0)	74 (56.9)	248 (54)	0.053^g^
Compliant to treatment schedule	160 (88.4)	125 (82.8)	111 (84.7)	396 (85.5)	0.334^g^

This table compares clinical characteristics between GLP-1 RA medication groups. No significant differences were found in BMI between groups. On average patients on Tirzepatide lost significantly greater weight in a month and a year compared to those on Liraglutide and Semaglutide, no significant differences were found between Semaglutide and Liraglutide. Duration of use significantly varied across treatment group with Tirzepatide users had the lowest duration of use < 1 year.

^a^Missing data for n = 9 and n = 18 removed. ^b^Missing data for n = 3. ^c^Missing data for n = 3. ^d^Missing data for n = 9 and n = 18 removed. ^e^Significance (p < 0.05). ^f^One-Way ANOVA (p < 0.05). ^g^Chi-Square Test (p < 0.05). ^*^p < 0.05.

Monthly weight loss varied significantly among groups (*p* = 0.002), with Tirzepatide users reporting losing the most weight (4.76 ± 2.82 kg), followed by Liraglutide (4.00 ± 2.41 kg) and Semaglutide (3.75 ± 2.21 kg). No significant differences in weight loss per month were found between the Semaglutide and Liraglutide groups (*U* = 9,502, *p* = 0.369; Table 2).

Tirzepatide users reported significantly greater weight in a year (8.48 ± 4.04 kg, *p* < 0.001) when compared to both groups. Semaglutide users reported more weight loss in a span of a year (7.14 ± 4.04 kg, *p* = 0.006) when compared to Liraglutide users (6.18 ± 3.83 kg). Data entries for *n* = 55 participants were missing for the weight loss per month survey question and *n* = 18 participant data was missing for the weight loss per year question.

Duration of use was significantly different across treatment groups. Tirzepatide users had a significantly higher proportion (88%, *p* = 0.002) in the shortest duration of use < 1 year category, while Liraglutide users had the largest proportion (10.4%) in the longest duration category of > 2 years. No significant differences were found in adherence to a diet during treatment, incidence of side effects, and compliance to treatment schedule across the three treatment groups. Consultation with a dietitian during treatment was the highest among the Semaglutide group (58.7%), however no significant differences were found (*p* = 0.053).

### 3.3 Side effects

Most side effects were reported similarly across groups, except the incidence of belching and anxiety (Table 3). Belching was significantly more commonly reported as a side effect among the Tirzepatide group (14.8%), compared to Semaglutide (4.4%), and Liraglutide (9.9%) users (*p* = 0.006). Anxiety was significantly (*p* < 0.001) more common among the Liraglutide group (23%) when compared to Semaglutide (8.8%) and Tirzepatide (11.1%) groups. Liraglutide users were significantly more likely to switch medications (40%, *p* < 0.001) compared to other groups. Participants could select multiple side effects, and 81 participants (17%) provided open-ended responses, which may influence interpretation.

**Table 3:** Comparison of side effects and treatment continuity between GLP-RA medication groups n = 468^a^.

**Side effect**	**Semaglutide (Ozempic or Wegovy) *n* = 181**	**Liraglutide (Saxenda or Victoza) *n* = 152**	**Tirzepatide (Mounjaro) *n* = 135**	**Total *n* = 468**	***P*−*value*^b^**
No side effects	17 (9.4)	11 (7.2)	13 (9.6)	41 (8.8)	0.719
Belching	8 (4.4)	15 (9.9)	20 (14.8)	43 (9.2)	0.006^*^
Loss of appetite	71 (39.2)	62 (40.8)	56 (41.5)	189 (40.4)	0.915
Constipation	51 (28.2)	39 (25.7)	44 (32.6)	134 (28.6)	0.425
Indigestion	19 (10.5)	15 (9.9)	11 (8.1)	45 (9.6)	0.771
Dizziness	72 (39.8)	69 (45.4)	59 (43.7)	200 (42.7)	0.566
Gallstones	6 (3.3)	7 (4.6)	3 (2.2)	16 (3.4)	0.553
Injection site reaction	6 (3.3)	13 (8.6)	10 (7.4)	29 (6.2)	0.112
Anxiety	16 (8.8)	35 (23)	15 (11.1)	66 (14.1)	< 0.001^*^
Headache	63 (34.8)	66 (43.4)	41 (30.4)	170 (36.3)	0.062
Stomach pain	46 (25.4)	53 (34.9)	34 (25.2)	133 (28.4)	0.100
Changed GLP-1	24 (13.4)	61 (40.1)	25 (18.8)	110 (23.7)	< 0.001^*^
If you suffered from side effects but still lost weight would you stop taking these injections?	82 (46.1)	75 (50)	48 (37.5)	205 (45)	0.105

When comparing side effects, no significant differences were found between the majority of side effects, except the incidence of belching and anxiety. Belching was significantly more common as a side effect among the Tirzepatide group (p = 0.006). Anxiety was significantly more common among Liraglutide group (p < 0.001). Lastly, 40% of Liraglutide users reported changing their GLP-1 RA medication.

^a^Missing data for n = 59 and n = 18 removed. ^b^Chi-Square Test significance p < 0.05. ^*^p < 0.05.

### 3.4 Satisfaction and quality of life

Satisfaction and satisfaction levels significantly varied across the three treatment groups. Tirzepatide users reported the highest satisfaction with treatment (88.1%, *p* < 0.001) (Table 4) and had significantly higher average satisfaction scores (2.66 ± 0.69, *p* < 0.001) compared to other groups. Tirzepatide users were also significantly more likely to recommend GLP-1 RA treatment (83.7%, *p* < 0.001). Quality of life improved significantly more for Tirzepatide users (60.6%, *p* < 0.001) compared to the other groups. In contrast, Liraglutide users reported the highest rate of worsening quality of life (33.9%). However, missing data (*n* = 111) may affect the interpretation of these findings, particularly as Tirzepatide had the smallest sample size (*n* = 99).

**Table 4:** Comparison of satisfaction, psychological health, and quality of life between GLP-RA medication groups *n* = 468^a^.

**Quality of life outcomes**	**Semaglutide (Ozempic or Wegovy) *n* = 181**	**Liraglutide (Saxenda or Victoza) *n* = 152**	**Tirzepatide (Mounjaro) *n* = 135**	**Total *n* = 468**	***P*−*value*^b^**
***N*** **(%)**					
Satisfied	142 (78.5)	99 (65.1)	119 (88.1)	360 (76.9)	< 0.001^*^
Satisfaction level (mean ± SD)	2.48 ±.067	2.25 ±.081	2.66 ±.069	2.44 ±.899	< 0.001^c*^
GLP-1 RA affected psychological health	73 (41.7)	80 (53.1)	59 (46.1)	212 (46.9)	0.096
Would recommend GLP-1 RA	128 (72.7)	77 (51.3)	108 (83.7)	313 (68.8)	< 0.001^*^
Reached goal	63 (35.4)	51 (34)	51 (39.)	165 (36.1)	0.611
**Quality of life**	***N*** = **146**	***N*** = **112**	***N*** = **99**	***N*** = **357**	
Improved quality of life	75 (51.4)	39 (34.8)	60 (60.6)	174 (48.7)	0.001^*^
No change	44 (30.1)	35 (31.3)	23 (23.2)	102 (28.6)	
Negatively affected quality of life	27 (18.5)	38 (33.9)	16 (16.2)	81 (22.7)	

When comparing satisfaction levels, Tirzepatide group were significantly more satisfied with treatment and results, suggesting this group perceived a better experience and outcome. Additionally, Tirzepatide users were significantly more likely to recommend treatment to friends and family when compared to other groups. Lastly, quality of life significantly improved for Tirzepatide, while it significantly worsened for the Liraglutide group.

^a^Missing data for n = 59 and n = 18 removed. ^b^Chi-Square Test significance p < 0.05. ^c^One-Way ANOVA (p < 0.05). ^*^p < 0.05.

## 4 Discussion

### 4.1 Main findings

The aim of this cross-sectional study was to examine the differences in weight-loss, side effects, and quality of life of patients taking different GLP-1 RA drugs. A total of 468 participants were included in the analysis of this study, with 39% on Semaglutide, 33% on Liraglutide, and 29% on Tirzepatide. Tirzepatide users experienced significantly greater weight loss and reported improvements in quality of life compared to users of other GLP-1 RA medications. Additionally, factors such as duration of use and side effects were analyzed to provide context for observed weight loss differences. Notably, 88% of Tirzepatide users had the shortest duration of use (< 1 year), while 10% of Liraglutide users had the longest duration of use (>2 years).

Certain side effects were more prevalent among specific medication groups. Belching was more commonly reported by Tirzepatide users, and anxiety was more commonly reported by Liraglutide users (Table 3). Lastly, Tirzepatide users reported significantly higher satisfaction levels, improvement on quality of life, and a higher likelihood of recommending treatment compared to the other medication groups (Table 4).

### 4.2 Comparison to other studies

This study's findings align with previous research demonstrating superior efficacy of Tirzepatide compared to both Semaglutide and Liraglutide. A systematic review of nine studies, seven of which were randomized-control trials, found that Tirzepatide resulted in significantly greater weight-loss, with an average difference of 3.78 kg compared to both Semaglutide and Liraglutide (21). Additionally, Semaglutide induced greater weight loss compared to Liraglutide, by a difference of 6.08 kg (21). These results align with the weight-loss per year results of this study, Semaglutide users reported significantly greater weight-loss per year than Liraglutide (Table 2). However, other factors must be considered when interpreting the results, for example, in this study, Tirzepatide users were the smallest sample participant group and had the shortest duration of use. Initially GLP-1 RAs demonstrate rapid rates of weight-loss in obese and overweight patients, due to initial metabolic adaptations to the medication that enhances its effect. However, studies suggest that the rate of weight-loss tends to plateau over time as the body adapts to the medications metabolic changes (16, 22, 23). Therefore, the significantly shorter duration of use among Tirzepatide users may explain their observed greater weight-loss outcome compared to Semaglutide and Liraglutide groups.

A comparative analysis including 53 clinical trials on GLP-1 RAs found Tirzepatide to be more effective compared to both Semaglutide and Liraglutide in reducing both body weight by an average difference of −8.47 kg and waist circumference by −6.77 cm (19). Additionally, Tirzepatide was found to be more effective for reducing hemoglobin A1C by a mean difference −2.10% and fasting blood glucose by −3.12 mmol/L compared to both Semaglutide and Liraglutide. However, Semaglutide was far more effective at clinically reducing low-density lipoprotein cholesterol (−0.16 mmol/L) and total cholesterol (−0.48 mmol/L) compared to other GLP-1 RAs which is a clinical element that was not analyzed in this study (19).

One proposed explanation of the enhanced efficacy of Tirzepatide is its dual mechanism of action (24). In addition to being a GLP-1 RA, Tirzepatide also acts as gastric inhibitory polypeptide (GIP) receptor agonist, allowing it to activate another signaling pathway that further enhances insulin sensitivity and appetite suppression, promoting weight loss (24).

Other potential factors that may affect weight-loss outcomes were examined in this study. No significant differences in BMI, diet adherence, compliance to treatment schedule, or the proportion of participants consulting with a dietitian were found between GLP-1 RA groups. This suggests that the differences observed were likely due to the GLP-1 RA used rather than external behavioral factors influencing weight loss.

### 4.3 Conclusion

This research delivers important knowledge regarding the effectiveness of GLP-1 RAs in weight loss among Kuwaiti patients with a specific attention to Semaglutide, Liraglutide, and Tirzepatide. Users of these medications exhibited substantial variability in weight loss results as well as adverse effects and life quality. Tirzepatide users achieved the most significant weight loss results both on a monthly and yearly basis and reported superior satisfaction levels along with better quality of life improvements. The brief usage period among Tirzepatide users potentially accounts for their significant weight loss through fast metabolic changes upon starting the medication.

Patients taking Liraglutide experienced greater anxiety and frequently changed their medication which indicates they may have suffered more adverse effects or were dissatisfied with their treatment. Semaglutide users experienced moderate weight loss and satisfaction while reporting fewer side effects than users of Tirzepatide and Liraglutide.

According to this study, side effects along with treatment costs and inability to achieve weight loss targets were the main factors leading to treatment discontinuation among all participant groups (Table 5). The study showed that 47% of participants discontinued treatment which demonstrates that despite GLP-1 RAs being effective weight loss agents, their long-term effectiveness is undermined by side effects and financial barriers (Table 5).

**Table 5:** Patients who stopped treatment, long-term side-effects, and reasons for stopping across treatment groups *n* = 220^a^.

***N* (%)**	**Semaglutide (Ozempic or Wegovy)**	**Liraglutide (Saxenda or Victoza)**	**Tirzepatide (Mounjaro)**	**Total**	***P*−*value*^b^**
Stopped medication	71 (39.89)	99 (66.44)	50 (37.87)	220 (47)	
Trouble eating after medication	27 (38.6)	24 (24.5)	16 (11)	67 (30.7)	0.146
**Reasons for stopping** ***N*** **(%)**					
Side effects	30 (42.3)	51 (34)	10 (20)	78 (35.5)	
Cost	7 (9.9)	22 (43.1)	22 (44)	51 (23.2)	
Reached my goal	25 (35.2)	29 (29.3)	18 (36)	72 (32.7)	
I found an alternative	9 (12.7)	10 (10.1)	0	19 (8.6)	

This table summarizes data for participants who have stopped treatment along with reasons for stopping treatment. Among the 468 participants, 220 or 47% of participants have ended their treatment. No significant differences were found between medication groups and long-term trouble eating after ending medication treatment. The most common reason for stopping treatment were because of side effects, followed by reaching goal, and cost, and lastly, I found an alternative.

^a^A total of n = 220 stopped treatment. ^b^Chi-Square Test significance p < 0.05.

### 4.4 Strengths and limitations

To our knowledge, this pilot study is the first to compare the differences in weight loss, side-effects, and quality of life of three popular GLP-1 RA injections used in Kuwait. The large number of participants (*N* = 468) included in the analysis of this study increases the reliability of the results. However, the cross-sectional nature of this study limits the ability to establish casual relationships and generalize its findings beyond this population. Although the survey design did use a validated instrument, the questions were a combination of both quantitative and qualitative, enriching the data and providing deeper insights into this understudied population. Additionally, the survey incorporated both quantitative and qualitative questions, providing a more comprehensive understanding of patient experiences. Factors such as motivation for weight loss, dietary adherence, and consultation with a dietitian were also considered, offering valuable context for interpreting the results.

Despite these strengths, this study is not without limitations. The reliance on self-reported data introduces recall bias further limiting reliability of the results and generalizability of conclusions to other populations. Other limitations include survey design, sample size, and variation in time since GLP-1 RA treatment. Specifically, this survey lacked validated tools to adequately measure outcomes and allowed participants to skip questions leading to missing data across variables. This sample had an uneven distribution of participants across medication groups and a significant variation in treatment duration. Approximately half (47%) of participants have discontinued treatment at the time of the survey, which may increase recall bias and reliability of reported outcomes. Additionally, drug concentrations used by participants were difficult to account for. GLP-1 RAs have different doses and are typically increased during treatment, making it difficult for participants to note one concentration used. Forming an accurate comparison between GLP-RA medications proved to be difficult in this population as participants reported changing medications during treatment, specifically among Liraglutide users. Lastly, the study population was overwhelmingly female (483 participants, 89%) limiting the generalizability of the findings to male patients.

### 4.5 Future implications and research directions

While this study provides valuable insights into the use of GLP-1 RA medications for weight-loss in Kuwait, the limitations of this study highlight the need for future research. Larger prospective cohort studies, tracking patients before during, and after treatment would provide a more comprehensive understanding of the long-term effects of GLP-1 RAs. Additionally, longitudinal studies are needed to assess weight-loss maintenance and determine whether maintenance doses are required for sustained effects. Future research should also explore clinical outcomes, such as hemoglobin A1C and lipid profiles, to tailor GLP-1 RA drug prescription based on the patient's individual needs and desired outcomes. Comparative studies between GLP-1 RA treatment and bariatric surgery may provide further insight into which approach yields superior health outcomes and improvements in quality of life. Lastly, studies focusing on the nutritional knowledge and dietary behaviors of GLP-1 RA patients could inform nutrition interventions and improve patient outcomes. Understanding how dietary habits interact with GLP-1 RA therapy may help optimize treatment, reduce side-effects, and support long-term weight management.

## Data Availability

The datasets presented in this article are not readily available as they contain secure personal data. Requests to access the datasets should be directed to saleh.ali2@ku.edu.kw.
